# The relationship between early and recent life stress and emotional expression processing: A functional connectivity study

**DOI:** 10.3758/s13415-020-00789-2

**Published:** 2020-04-28

**Authors:** Andrzej Sokołowski, Monika Folkierska-Żukowska, Katarzyna Jednoróg, Craig A. Moodie, Wojciech Ł. Dragan

**Affiliations:** 1 Department of Neurology, Memory and Aging Center, UCSF Weill Institute for Neurosciences, University of California, San Francisco, CA USA; 2 The Interdisciplinary Centre for Behavioral Genetics Research, Faculty of Psychology, University of Warsaw, Warsaw, Poland; 3 Laboratory of Language Neurobiology, Nencki Institute of Experimental Biology, Polish Academy of Sciences, Warsaw, Poland; 4 Department of Psychology, Stanford University, Stanford, CA USA

**Keywords:** Stress, Faces, Emotional expression, Mismatch hypothesis, Cumulative stress, Functional connectivity

## Abstract

The aim of this study was to characterize neural activation during the processing of negative facial expressions in a non-clinical group of individuals characterized by two factors: the levels of stress experienced in early life and in adulthood. Two models of stress consequences were investigated: the match/mismatch and cumulative stress models. The match/mismatch model assumes that early adversities may promote optimal coping with similar events in the future through fostering the development of coping strategies. The cumulative stress model assumes that effects of stress are additive, regardless of the timing of the stressors. Previous studies suggested that stress can have both cumulative and match/mismatch effects on brain structure and functioning and, consequently, we hypothesized that effects on brain circuitry would be found for both models. We anticipated effects on the neural circuitry of structures engaged in face perception and emotional processing. Hence, the amygdala, fusiform face area, occipital face area, and posterior superior temporal sulcus were selected as seeds for seed-based functional connectivity analyses. The interaction between early and recent stress was related to alterations during the processing of emotional expressions mainly in to the cerebellum, middle temporal gyrus, and supramarginal gyrus. For cumulative stress levels, such alterations were observed in functional connectivity to the middle temporal gyrus, lateral occipital cortex, precuneus, precentral and postcentral gyri, anterior and posterior cingulate gyri, and Heschl’s gyrus. This study adds to the growing body of literature suggesting that both the cumulative and the match/mismatch hypotheses are useful in explaining the effects of stress.

**Electronic supplementary material:**

The online version of this article (10.3758/s13415-020-00789-2) contains supplementary material, which is available to authorized users.

## Introduction

Emotional facial expressions carry important social information and can excite emotions in other people (Campos, Frankel, & Camras, 2004). Efficient recognition and processing of emotional facial expressions facilitate social communication (Frith, 2009). Both acute (van Marle, Hermans, Qin, & Fernández, 2009) and past stress (Nusslock & Miller, 2016; Pechtel & Pizzagalli, 2011), as well as various psychiatric conditions (Joormann & Gotlib, 2007; Larøi, Fonteneau, Mourad, & Raballo, 2010; Masten et al., 2008) may alter emotional processing, including the processing of facial expressions.

Processing of emotional expressions involves the activation of a broad neural network (Bernstein & Yovel, 2015). The fusiform face area (FFA), occipital face area (OFA), and posterior superior temporal sulcus (pSTS) are important regions responsible for face detection and play an essential role in the processing of emotional expressions (Duchaine & Yovel, 2015; Haxby, Hoffman, & Gobbini, 2000). While the fusiform gyrus is activated in response to face stimuli, regardless of facial expression (Tong, Nakayama, Moscovitch, Weinrib, & Kanwisher, 2000), it is also involved in the processing of emotional expressions (Ganel, Valyear, Goshen-Gottstein, & Goodale, 2005). Indeed, a recent review has confirmed that there is an overlap between facial identification and expression processing, and that both the FFA and pSTS are particularly engaged in these processes (Lander & Butcher, 2015). However, the processing of emotional facial expressions also engages an extensive neural network associated with emotional processing (Duchaine & Yovel, 2015; Haxby et al., 2000). Fusar-Poli, Placentino, Carletti, Allen, et al. (2009a), in their meta-analysis, reported increased reactivity to emotional faces in the fusiform gyrus, middle temporal gyrus (MTG), middle occipital gyrus (MOG), and subcortical structures such as the amygdala, putamen, and caudate nucleus.

Adversities in early life can have a long-term impact on brain functioning which, in turn, often affects emotional processing. Hence, individuals who have experienced early adversities often exhibit altered neural activation in response to emotional faces. This is especially true for the activity of the amygdala, which is a structure heavily implicated in affective processing. Childhood maltreatment, institutionalization, caregiver deprivation, and negligence have been shown to be positively correlated with amygdala activation in response to both negative (Dannlowski et al., 2012; Maheu et al., 2010; McCrory et al., 2013; Tottenham et al., 2011) and positive facial expressions (McCrory et al., 2013; but see Taylor, Eisenberger, Saxbe, Lehman, & Lieberman, 2006). However, previous studies are inconsistent with regard to amygdala reactivity to emotional expressions. Although Garrett et al. (2012) and van Harmelen et al. (2013) found enhanced amygdala reactivity to emotional faces in adolescents and adults who experienced childhood maltreatment, other studies did not report differences in amygdala reactivity in adolescents (Colich et al., 2017) and adults (Jedd et al., 2015) with and without a history of early adversities. These inconsistent results may be a consequence of the fact that the developmental timing of unfavorable events can differently impact the functioning of the amygdalae (Tottenham & Sheridan, 2010). Moreover, the type of task may also impact the findings of a given study. Implicit and explicit processing of emotional stimuli involve distinct neural networks (Fusar-Poli, Placentino, Carletti, Landi, et al., 2009b; Phillips, Ladouceur, & Drevets, 2008). Processing of facial expressions is characterized by a more implicit regulation goal, while cognitive reappraisal requires more explicit and controlled regulation (Braunstein, Gross, & Ochsner, 2017). Stress may also impact the functioning of other brain regions involved in the processing of emotional facial expressions. For instance, Gollier-Briant et al. (2016) found a positive relationship between lifetime distress and adolescents' brain activation while viewing angry faces in the inferior frontal gyrus (IFG), middle frontal gyrus (MFG), orbitofrontal gyrus (OFC), MTG, and superior temporal gyrus (STG).

Life stress may lead to both dendritic and synaptic remodelling of brain regions involved in emotional processing (for review, see McEwen et al., 2015). This can contribute to altered functional connectivity – i.e., the way that the activity of distinct brain regions is correlated in time – during processing of emotional expressions. In a study using emotional facial stimuli with children and adolescents who had experienced institutionalization, a negative correlation was observed between the activity of the amygdala and medial prefrontal cortex (mPFC), which is normally characteristic of a more mature brain (Gee et al., 2013). In another study, positive correlation between the activity of the amygdala and the hippocampus, IFG, medial frontal gyrus, frontal pole, and inferior parietal lobule (IPL) was found during a face-matching task in adults who had experienced childhood maltreatment (Jedd et al., 2015). However, the impact of recent stress in early adulthood on the processing of emotional expressions is still unexplored, alhough acute stress has been shown to enhance activation in the early visual cortex and FFA during the processing of emotional facial expressions (van Marle et al., 2009).

The effects of stress on the brain largely depend on their severity and timing; most studies focus on early life stress, which, due to its occurrence during sensitive periods of neural development, leaves serious and long-lasting impacts (for review, see: De Kloet, Joëls, & Holsboer, 2005; McEwen et al., 2015; Tottenham & Sheridan, 2010). Yet, there is evidence that stress experienced later in life also has lasting consequences. Chronic exposure to stress hormones decreases one’s capacity to endure future stressors, impacting mental health (Lupien, McEwen, Gunnar, & Heim, 2009). In this paper, we examine how early stress and recent stress in early adulthood affect emotional processing; this is especially interesting given that both the cumulative effects of stress and an interaction between early and recent life stress have been linked to psychopathology (McLaughlin, Conron, Koenen, & Gilman, 2010; Nederhof, Ormel, & Oldehinkel, 2014). There are two approaches that can be used to describe the effects of early and recent stress on brain activation during the processing of emotional expressions: the cumulative stress hypothesis and the match/mismatch hypothesis (Levine, 2005; Nederhof & Schmidt, 2012).

The cumulative stress hypothesis assumes that the effects of stress are additive. According to this hypothesis, prior stressors lead to increased sensitivity to stressful experiences in the future, which is why it is also referred to as the sensitization hypothesis (Hammen, Henry, & Daley, 2000). Early adversities may decrease resilience, lower the activation threshold for subsequent stressors, and enable weaker stressors to trigger the stress response (Espejo et al., 2007). Stressful experiences are associated with memory, executive functions, emotion regulation, and the processing of social and affective stimuli deficits (Pechtel & Pizzagalli, 2011). Moreover, chronic exposure to stress may lead to neurodevelopmental alterations within the structure and activity of the brain (McEwen & Gianaros, 2011).

On the other hand, the match/mismatch hypothesis assumes that early adversities promote optimal coping with similar events in the future through fostering the development of coping strategies (Santarelli et al., 2014). This approach emphasizes the importance of the interaction between early life stress and recent stress. Hence, individuals with matched environments (i.e., similar levels of early life stress and recent stress) are better off than those with mismatched environments (i.e., different levels of early and recent stress; Nederhof & Schmidt, 2012). This model can be placed within the more general context of life history theory, which analyses the development of optimal coping strategies, maximizing an organism’s fitness to its external environment (Del Giudice, Gangestad, & Kaplan, 2015). This evolutionary approach takes into account prolonged periods of early life vulnerability and underlines the significance of psychological and biological adaptation. In particular, early life environmental harshness and unpredictability have been shown to be important factors influencing the development of life history strategies (Ellis, Figueredo, Brumbach, & Schlomer, 2009). Both unfavorable external environmental and internal (i.e., somatic) predictions can forecast future adversities, thus impacting the evolution of regulation strategies and the maximization of evolutionary fitness (Rickard, Frankenhuis, & Nettle, 2014). The match/mismatch hypothesis has mainly been tested in animal studies. For example, stress hormones improved long-term potentiation in dentate gyrus slices extracted from mice that experienced early maternal deprivation (while doing the opposite for control mice), suggesting that adversity in early life can prepare the brain for better functioning under stress (Oomen et al., 2010). Few studies have used this approach in the context of the consequences of stress in humans (Nederhof et al., 2014; Paquola, Bennett, Hatton, Hermens, & Lagopoulos, 2017; Sandman, Davis, & Glynn, 2012).

Based on these hypotheses, it is possible to create and test two models of the influence of stress on the brain, without necessarily assessing whether or not the outcomes are adaptive. The only MRI study establishing a relationship between these two approaches regarding the impact of stress on the brain suggests that both cumulative effects and match/mismatch effects may occur in parallel, affecting different domains of brain structure and function in the resting state (Paquola et al., 2017). However, no study has yet looked into these two hypotheses in the context of task-related neural activation during emotional processing. Thus, the aim of this study was to characterize the neural activation during processing of negative facial expressions in non-clinical groups of individuals characterized by two factors: the levels of stress experienced in early life and in adulthood. This design allows the testing of both the cumulative stress model and the match/mismatch model in the context of neural correlates of emotional expression processing. This may help elaborate the usefulness of the two approaches to explaining the effects of stress on functional connectivity during the processing of emotional facial stimuli. The interaction between early life stress and recent stress in adulthood would indicate that functional connections between structures associated with face processing depend on the match/mismatch model. Observing a relationship between the cumulative effects of stress and brain activation or functional connectivity between certain structures during face processing would indicate that the effects of stress on the activations/connections of these structures is additive. It is possible that both models will provide significant results, but involving different structures or connections. Since our investigations were performed using an emotional expression paradigm, we anticipated observing effects on the neural circuitry of structures engaged in face perception and emotional processing (i.e., the amygdala, FFA, OFA, and pSTS). The match/mismatch model was examined using regression analysis with main and interaction effects. We hypothesized that early life stress would be related to increased functional connectivity between the amygdala, FFA, OFA, and pSTS and regions in the prefrontal cortex, visual cortex, and other parts of the face-processing system (Herringa et al., 2016; Lieslehto et al., 2017), while recent stress in adulthood would be associated with increased connectivity with the amygdala, prefrontal cortex, and FFA (Li, Weerda, Milde, Wolf, & Thiel, 2014; Reynaud et al., 2015). As the match/mismatch model has mainly been tested in animal studies and little is known about the interaction between early and recent life stress, we did not formulate a priori hypotheses regarding brain structures involved in the neural circuitry for this interaction. Based on previous studies on long-term consequences of stress, we also hypothesized that cumulative stress would be related to increased functional connectivity between the amygdala, FFA, OFA, and pSTS with the prefrontal cortex, hippocampus, and amygdala (Jedd et al., 2015; Lupien et al., 2009).

## Material and methods

### Participants

A total of 85 (42 females) young adults (aged 19–25 years, *M* = 21.64; *SD* = 1.82) took part in the study. Of the 90 recruited participants, one was rejected due to MRI contraindication, one did not finish the task, and data from an additional three subjects were discarded due to insufficient coverage of the amygdala. Participants were selected from a community sample (*N* = 503) based on Early Life Stress Questionnaire (ELSQ; Cohen et al., 2006) and Recent Life Changes Questionnaire (RLCQ; Rahe, 1975) outcomes, as measures of stressful events. The selection was based on the quartiles of the variables’ distributions in the community sample. First, participants with the lowest (*n* = 22) and highest (*n* = 21) levels of stress were selected for the study. Second, we selected participants with low levels of stress in childhood and high levels of stress in adulthood (*n* = 20), as well as those with high levels of stress in childhood and low levels of stress in adulthood (*n* = 22). Low levels of stress in childhood and adulthood were operationalized as low scores on ELSQ and RLCQ (below the first quartile), while high levels of stress as high scores (above the fourth quartile). Exclusion criteria included the reported presence of neurological or psychiatric disorders, traumatic brain injury, addiction to alcohol, drugs, or other psychoactive substances, as well as any MRI contraindications. All participants provided written informed consent and were paid the equivalent of 60€ in local currency. The procedure was approved by the local ethics committee. The study was conducted in accordance with the guidelines of the Declaration of Helsinki.

### Assessment

Early life stress was assessed with the ELSQ (Cohen et al., 2006; Sokołowski & Dragan, 2017). It measures exposure to 19 stressful events, including emotional, sexual, and physical abuse, violence, negligence, parental divorce, surgery, parental death, separation, etc. Participants reported whether they had experienced any of these events before the age of 12 years, with a maximum score of 19. The most frequently reported events were: emotional abuse, severe family conflict, domestic violence, and being bullied. Cronbach’s α reliability index for this measure in our sample was equal to .85.

The RLCQ (Rahe, 1975; Sobolewski, Strelau, & Zawadzki, 1999) was used to measure levels of stress in adulthood. It includes both negative and positive (yet stressful) events, such as a death in the family, accident, losing a job, marriage, the birth of a child, moving to a different city, etc. Participants were asked whether they had experienced stressful events in the previous 24 months, with a maximum score of 73. The most frequently reported events on the questionnaire were: beginning or ceasing school or college, a change in family get-togethers, a change in usual type and amount of recreation, a change in personal habits, and work-related changes (new type of work, changed hours and conditions, or more responsibilities). Cronbach’s α reliability index for this measure in our sample was equal to .94.

The Socioeconomic Status Index was computed based on the Scale of Material Remuneration (Domański, Sawiński, & Słomczynski, 2009), which covers average income related to the participants’ profession.

### Experimental task

Participants were tested in an fMRI scanner during a single session. Subjects were familiarized with the scanner and the experimental task procedure in a mock scanner with stimuli different from those used in the experiment. Participants performed three tasks as part of a larger project, and the data from the face-matching task are presented here. A set of 150 color face images of 30 individuals was used. The stimuli were taken from a standardized database (Warsaw Set of Emotional Facial Expression Pictures; Olszanowski et al., 2015). Pictures displayed different emotions (fear, anger, disgust, and happiness) as well as neutral faces. Only negative emotional expressions were examined in this study.

**Fig. 1:**
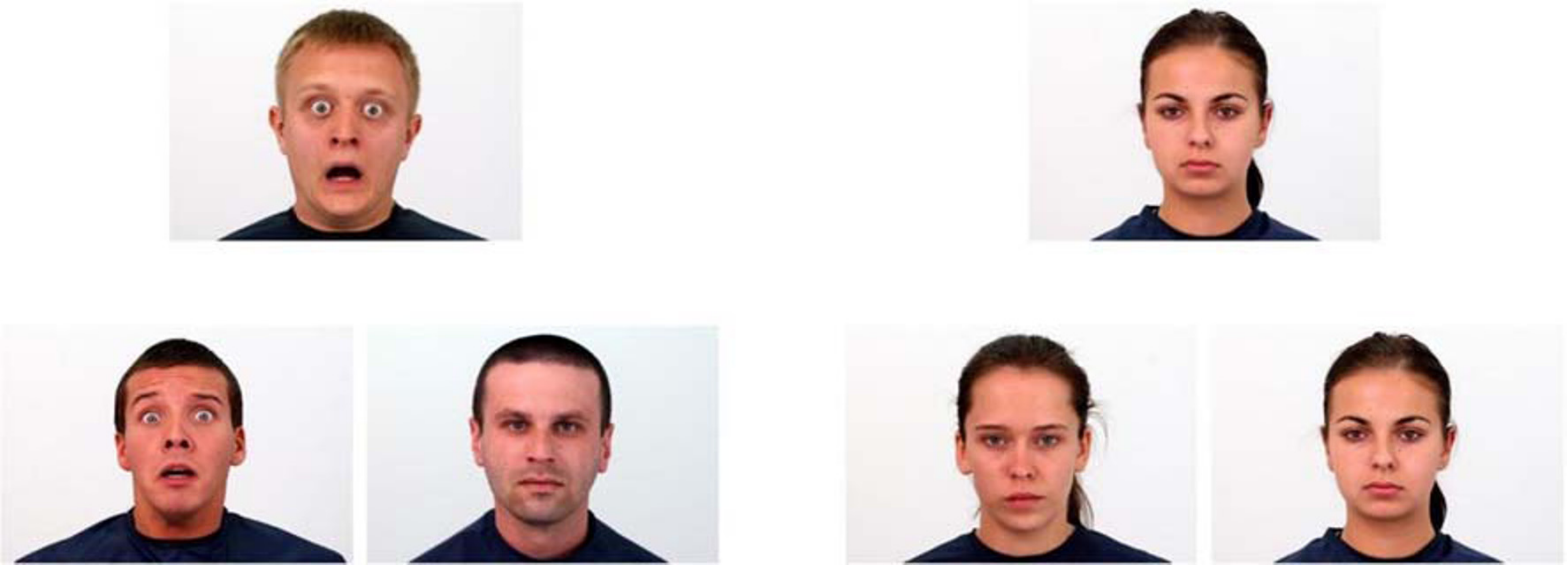
Face-matching task – emotional (**left**) and control (**right**) conditions. The emotional and control blocks were presented in an alternating order; six trials of the same emotional condition within each block were used

The face-matching task was adapted with adjustments from previous studies on emotion recognition and processing (Åhs et al., 2014; Hariri, Bookheimer, & Mazziotta, 2000). Two conditions were used to measure neural response to facial expressions: emotional and control. During each trial, three faces were presented on the screen – one at the top and two at the bottom. In the emotional condition, participants were asked to match the emotional expression – i.e., to select the face on the bottom that expressed the same emotion as the target face on top (Fig. 1). In the control condition, all faces were neutral and subjects were instructed to select which of the bottom pictures was the same as the target face on top. Although geometric shapes are usually used as a sensorimotor control (Åhs et al., 2014; Demers, Drabant Conley, Bogdan, & Hariri, 2016; Goetz et al., 2014), we decided to use different stimuli in the control condition. Neutral faces were chosen to subtract the activation related to face perception in predefined contrasts, leaving only activation related to the processing of emotional expressions.

The stimuli were presented in a block design with two conditions: emotional and neutral. Each block started with an instruction slide (4 s) followed by six trials (three female and three male face trios, presented randomly). Images were presented for 4 s in a randomized fashion for all conditions, with a variable inter-stimulus interval of 1.5–3 s. Each of the two runs consisted of four experimental (emotional) blocks (fear, anger, disgust, and happiness; displayed randomly) and four control blocks, presented alternately. Stimuli were presented against a gray background using the Presentation software (Neurobehavioral Systems, Inc., Berkeley, CA). Accuracy and response time were registered as behavioral measures as they have been shown to be indicators of emotion recognition and bias in emotional processing (Masten et al., 2008).

### Behavioral analysis

In order to examine the effects of early life stress in childhood and recent stress in adulthood on reaction time (RT) and accuracy, regression analyses were performed for RT and accuracy. To remove variance unrelated to emotion processing, RT and accuracy indices were calculated, in-line with the approach used in neuroimaging contrasts. To this end, mean RT in the neutral condition was subtracted from mean RT in the negative conditions (fear + anger + disgust), and accuracy in the neutral condition was subtracted from accuracy in the negative conditions. The main effects of early life stress, recent stress, and their interaction were examined.

To test whether cumulative stress was a predictor of RT and accuracy, regression analyses were performed with cumulative stress index as a predictor – one with RT index as the dependent variable and one with accuracy index as the dependent variable.

### MRI data acquisition and preprocessing

Whole-brain functional and structural images were acquired using a 3T MRI scanner (Trio TIM, Siemens, Germany) equipped with a 32-channel head coil. First, a localizer and high-resolution T1-weighted images were obtained. TR/TI/TE = 2,530/1,100/3.32 ms; flip angle = 7°; PAT factor = 2; FoV = 256 mm; voxel dimensions = 1 mm isotropic; 256 × 256 voxel resolution. Functional images were acquired using a T2-weighted, gradient-echo echo planar imaging (EPI) pulse sequence during two functional runs. For each experimental run, 176 whole-brain volumes were recorded with the following parameters: TR/TE=2000/30 ms; flip angle = 90°; 64 × 64 matrix size; FoV = 224 mm; 3.5×3.5 mm vox size; 35 slices (interleaved ascending); 3.5-mm slice thickness.

Analyses were performed using Statistical Parametric Mapping (SPM12; Wellcome Department of Cognitive Neurology, London, UK), implemented in MATLAB (2018; The MathWorks Inc., Natick, MA, USA). Images were spatially realigned, slice-time corrected (to the middle slice), co-registered to the first functional image, segmented, normalized to the standard Montreal Neurological Institute (MNI) template, and spatial smoothed with an 8-mm isotropic Gaussian kernel.

### First-level analysis

Data from two experimental runs were modeled with a general linear model (GLM) for each subject for the first-level analysis. Vectors representing all task conditions and instructions were added to the model: (a) fearful faces; (b) angry faces; (c) disgusted faces; (d) happy faces; (e) neutral faces; and (f) instructions. Each regressor was convolved with a canonical hemodynamic response function. The model included regressors of no interest to account for head motion. Additionally, the Artifact Detection Tool (ART) toolbox was used to determine motion-affected volumes exceeding 2 mm or 0.05 rad movement thresholds (which were excluded from analyses).

### Second-level analysis

Single-subject contrasts (fearful, angry, and disgusted faces > neutral faces, i.e., a [-1 -1 -1 3] contrast) underwent second-level analyses. To explore the match/mismatch model, a regression model with standardized scores of early life stress and recent life stress and their interaction (computed as the multiplication of standardized early and recent stress scores) was used at the group level. To test the cumulative stress hypothesis, the cumulative stress index was used in a regression analysis. This index was computed as a sum of standardized early and recent stress scores. Family-wise error (FWE < .05) was used to control for multiple comparisons in whole-brain analysis.

### gPPI analysis

The CONN functional connectivity toolbox (v.18a; http://www.nitrc.org/projects/conn) with a false discovery rate (FDR) correction was used to conduct the functional connectivity analysis. The analyses were corrected for multiple comparisons for all eight seeds, that is a *p* < .001; FDR < .00625 threshold (*p*-value for cluster) was used. Functional data were denoised with use of the respective T1-weighted images normalized to the MNI space, with five regressors for white matter, five regressors for cerebrospinal fluid, and movement parameters from ART toolbox. The acceptance threshold for the denoised signal (voxel to voxel correlation) was on average r < .1. All regressors of interest (i.e., fearful, angry, disgusted, and neutral faces) and no interest (i.e., happy faces, instructions, and motion parameters) were entered into a gPPI model, and a group-level seed-based connectivity analysis was performed for the negative emotional expressions > neutral faces contrast. To explore the match/mismatch model, a regression model with standardized scores of early life stress and recent life stress as well as their interaction was used at the group level. To test the cumulative stress hypothesis, a regression analysis with the cumulative stress index was performed. The functional connectivity scores between seeds and whole clusters were extracted using the CONN toolbox for each participant. Positive connectivity indicates positive Fisher-transformed correlation coefficient values between BOLD signals in the seed and a cluster (i.e., the two structures are likely to be active at the same time), while negative connectivity refers to negative Fisher-transformed correlation coefficient values between BOLD signals in the seed and a cluster (i.e., when one structure is active, the other one is not active and vice versa).

### Seed definition

The bilateral amygdala, FFA, OFA, and pSTS were chosen as seed regions based on their involvement in the processing of facial expressions: the amygdala is a crucial structure involved in emotional processing (Phelps & LeDoux, 2005; van Harmelen et al., 2013); the FFA (Ganel et al., 2005; Vuilleumier, Armony, Driver, & Dolan, 2001; Winston, O’Doherty, & Dolan, 2003; Xu & Biederman, 2010) and pSTS are involved in the processing of emotional facial expressions (Engell & Haxby, 2007; Fox, Moon, Iaria, & Barton, 2009; Ganel et al., 2005; Pitcher, Duchaine, & Walsh, 2014); the OFA was selected because of its involvement in face processing (Duchaine & Yovel, 2015; Haxby et al., 2000; Nagy, Greenlee, & Kovács, 2012).

Seeds were defined prior to data analysis. The amygdala has a clearly determined anatomical location, hence it was defined using the Automated Anatomical Labeling (AAL) atlas implemented in the Wake Forest University Pickatlas (WFU_PickAtlas 3.0.5). Because a face area localizer task (i.e., faces vs. non-faces contrast) was not used to localize the face-specific regions in our study, Activation Likelihood Estimate (ALE) meta-analysis was conducted to localize the FFA, OFA, and pSTS. They were localized based on loci from 30 recent studies concerning face processing (see Table S1 in Online Supplementary Material for the list of loci). Talairach coordinates were transformed to MNI coordinates using GingerALE (v.2.3.6; http://www.brainmap.org/ale/). All loci were entered into the GingerALE analysis with FDR < .05. The MarsBar toolbox was used to build region of interest (ROI) clusters. Spheres with 6 mm radii were built around the peak activation reported by ALE meta-analysis. The following ROIs (coordinates in MNI space) were defined: left FFA (-41 -52 -19), right FFA (42 -50 -20), left OFA (-39 -78 -10), right OFA (42 -77 -10), left pSTS (-52 -57 12), and right pSTS (53 -50 10). The Harvard-Oxford atlas was used to label the results.

## Results

### Behavioral results

There was no main effect of early life stress in childhood, recent stress in adulthood, nor their interaction on RT or accuracy. The cumulative stress index was a predictor of the RT index (F(1,83) = 4.14; *p* < .05), but not the accuracy index. Individuals with higher stress levels had lower RTs when processing negative expressions.

### Whole-brain analyses

Whole-brain analyses were conducted for the negative emotional expressions versus neutral faces contrast. First, analyses were performed across all participants (see Table S2 and Fig. S1 in the Online Supplementary Material). This revealed activations (*p* < .001 unc.; FWE < .05) mainly in the inferior and middle frontal gyri (IFG and MFG), fusiform gyrus (FuG), and inferior occipital gyrus (IOG). Activation in the bilateral amygdala did not survive FWE correction (*p* < .001; FWE > .05). There was deactivation in the anterior and posterior cingulate cortex (ACC and PCC), middle occipital gyrus (MOG), and superior frontal gyrus (SFG). Second, whole-brain analyses were performed for the match/mismatch model. Contrary to our prediction, there were no significant main effects of early and late life stress. The interaction was not significant, indicating no differences in whole-brain activation related to the match/mismatch model. Third, the cumulative model was tested, but revealed no significant cumulative effects of stress on whole-brain activation.

### Functional connectivity

#### Match/mismatch hypothesis

**Fig. 2:**
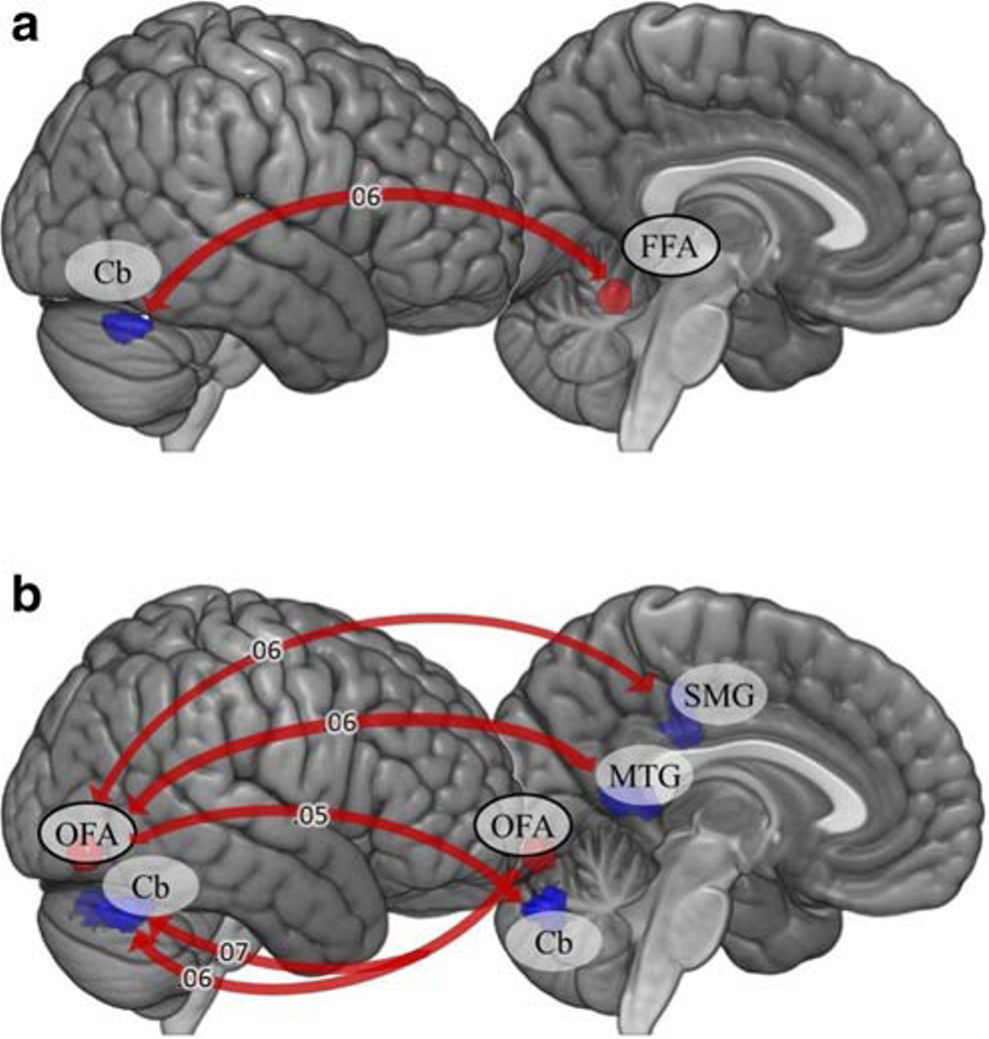
Seed-based functional connectivity during face-matching for the match/mismatch model. Arrows indicate the interaction between early and recent life stress for functional connectivity. Beta values, i.e., Fisher-transformed correlation coefficients (connectivity strength) are displayed on the arrows. (**A**) FFA seeds; (**B**) OFA seeds. *Cb* cerebellum, *FFA* fusiform face area, *MTG* middle temporal gyrus, *OFA* occipital face area, *SMG* supramarginal gyrus

**Fig. 3:**
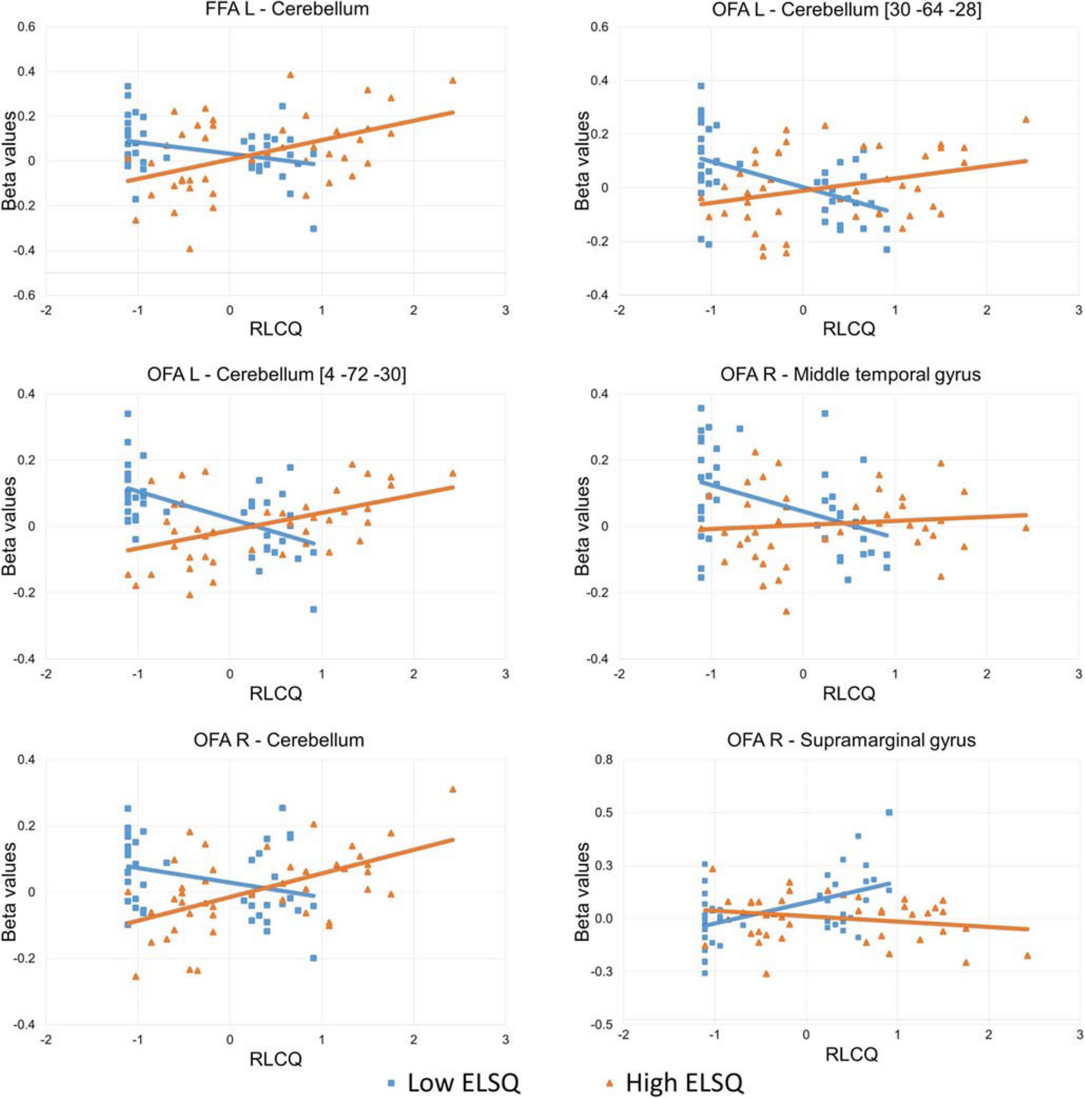
Plots representing seed-based functional connectivity strengths for the interaction between early and recent life stress. Beta values, i.e., Fisher-transformed correlation coefficients are presented. The sample was divided according to the median ELSQ score solely for illustrative purposes. ELSQ corresponds to early life stress, RLCQ corresponds to recent stress in adulthood. *ELSQ* Early Life Stress Questionnaire, *FFA* fusiform face area, *OFA* occipital face area, *RLCQ* Recent Life Changes Questionnaire

Results for the interaction between early and recent life stress in seed-based connectivity for given seeds are presented in Table 1 and Figs. 2 and 3. The main effects of early life stress and recent stress in adulthood are presented in Table 2.

**Table 1:** Regression analysis for the interaction between early and recent life stress in functional connectivity during the face-matching task

Seed	Brain region		Cluster size (voxels)	*p*-value for cluster (FDR)	β	x	y	z
FFA L	Cerebellum VI	R	170	.002	.06	30	-64	-28
OFA L	Cerebellum Crus I / Cerebellum VI	R	333	<.001	.07	30	-64	-28
	Cerebellum Crus I	R	276	<.001	.06	4	-72	-30
OFA R	Middle temporal gyrus	L	352	<.001	.06	-48	-52	8
	Cerebellum Crus I	L	216	<.001	.05	-28	-74	-32
	Supramarginal gyrus	L	172	.002	.06	-64	-28	44

Beta values, i.e., Fisher-transformed correlation coefficients (connectivity strength), for the interaction between early and recent life stress. Regions are defined by MNI coordinates; *L* left hemisphere, *R* right hemisphere

**Table 2:** Main effects of early and recent life stress on functional connectivity during the face-matching task

Seed	Brain region		Cluster size (voxels)	*p*-value for cluster (FDR)	β	x	y	z
Amygdala R	*Main effect of ES Negative correlation*							
	Central opercular	L	181	<.001	-.08	-48	-18	14
FFA R	*Main effect of ES Negative correlation*							
	SMA	LR	432	<.001	-.08	0	-2	62
pSTS R	*Main effect of RS Positive correlation*							
	Insula	R	355	<.001	.07	40	12	-8

Regions are defined by MNI coordinates. *ES* early stress, *FFA* fusiform face area, *L* left hemisphere, *pSTS* posterior superior temporal sulcus, *R* right hemisphere, *RS* recent stress

### Cumulative stress hypothesis

**Fig. 4:**
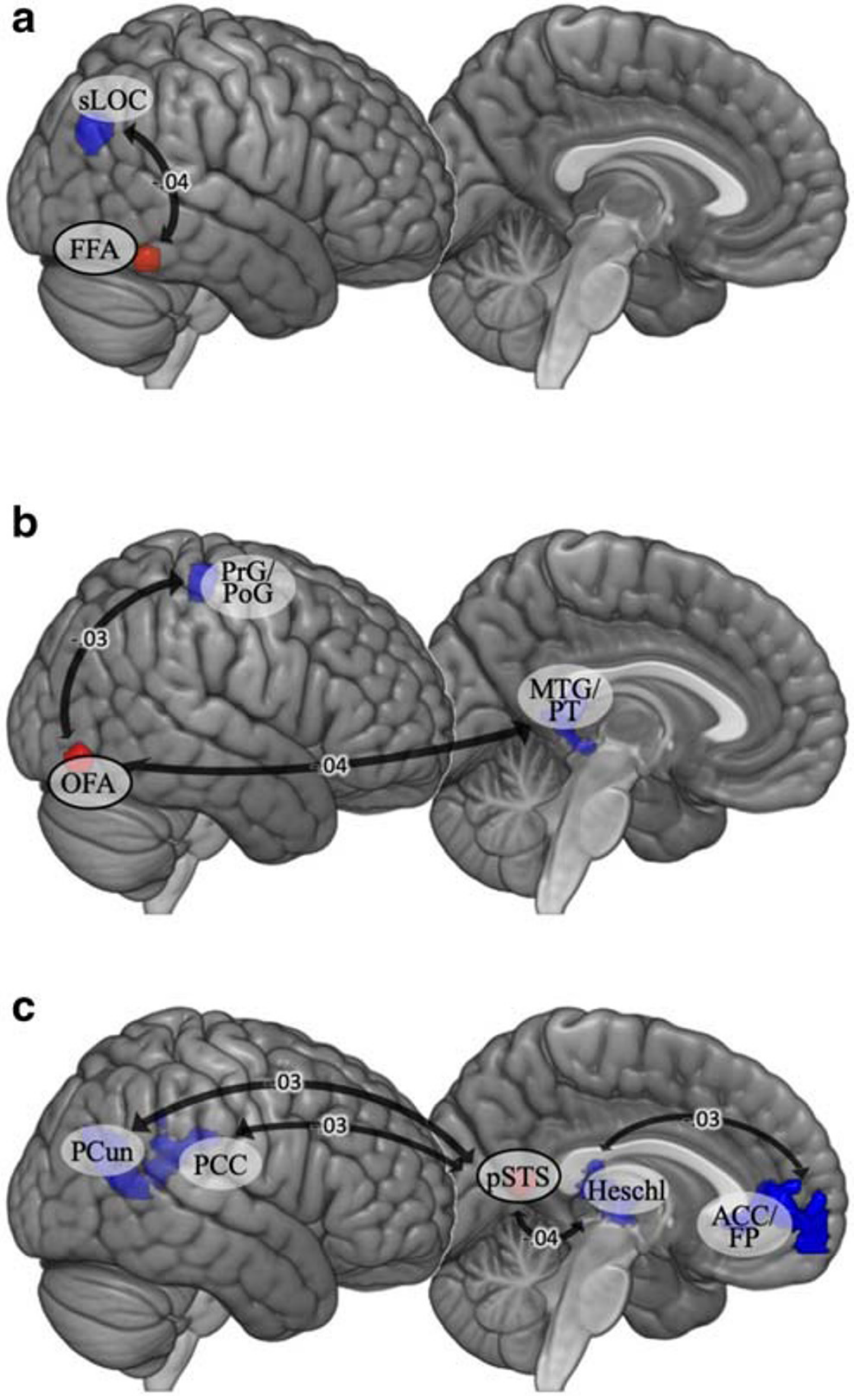
Seed-based functional connectivity during the face-matching task for the cumulative stress model: (**A**) FFA seeds; (**B**) OFA seeds; (**C**) pSTS seed. Beta values, i.e., expected increases in Fisher-transformed correlation coefficients (connectivity strength), for each unit-increase in cumulative stress levels are displayed on the arrows. *ACC* anterior cingulate cortex, *FFA* fusiform face area, *FP* frontal pole, *Heschl* Heschl’s gyrus, *MTG* middle temporal gyrus, *OFA* occipital face area, *PCC* posterior cingulate cortex, *PCun* precuneus, *PoG* postcentral gyrus, *PrG* precentral gyrus, *pSTS* posterior superior temporal sulcus, *PT* planum temporale, *sLOC* superior lateral occipital cortex

A seed-based connectivity analysis was used to examine neural circuitry related to cumulative stress. The results are reported in Table 3 and Fig. 4.

**Table 3:** Regression analysis for cumulative stress in functional connectivity during the face-matching task

Seed	Contrast and brain region		Cluster size (voxels)	*p-*value for cluster (FDR)	β	x	y	z
FFA R	Negative correlation							
	sLOC	R	182	.001	-.04	26	-68	32
OFA R	Negative correlation							
	MTG/PT	L	219	.001	-.04	-44	-40	10
	PrG/PoG	R	149	.003	-.03	18	-26	60
pSTS L	Negative correlation							
	ACC/FP	L	723	<.001	-.03	-6	28	-4
	PCun	R	541	<.001	-.03	24	-60	12
	PCC	R	431	<.001	-.03	6	-38	24
	Heschl's gyrus	L	421	<.001	-.04	-40	-26	10

Regions are defined by MNI coordinates. *L* left hemisphere, *R* right hemisphere, *ACC* anterior cingulate cortex, *FFA* fusiform face area, *FP* frontal pole, *MTG* middle temporal gyrus, *OFA* occipital face area, *PCC* posterior cingulate cortex, *PCun* precuneus, *PoG* postcentral gyrus, *PrG* precentral gyrus, *pSTS* posterior superior temporal sulcus, *PT* planum temporale, *sLOC* superior lateral occipital cortex

## Discussion

The present study investigated the impact of stress on brain circuitry related to the processing of negative emotional expressions. We examined two models of the consequences of stress based on the match/mismatch and cumulative stress hypotheses: the first hypothesis posits a beneficial effect associated with levels of stress in early and recent life being similar, even if these levels are high; the second suggests that stress has an additive negative effect. Both of these effects are assumed to have some underlying physiological mechanisms related to the HPA axis and the brain (De Kloet et al., 2005; McEwen et al., 2015). Previous studies have shown that, although the two stress consequence hypotheses seem mutually exclusive, models of stress impact on brain structure and function based on these hypotheses can, in fact, act in parallel. Depending on the structure or process, stress may have an impact that is better explained by the cumulative stress model or the match/mismatch model (Paquola et al., 2017; Zalosnik, Pollano, Trujillo, Suárez, & Durando, 2014). Thus, when talking about the neural correlates of experienced stress, it may be that one shouldn’t contrast these two models in terms of adaptiveness, but rather in terms of the different ways in which levels of stress throughout life can influence different structures and circuits in the brain. Here, we compare results for these two models in order to discuss potential differences in the impact of cumulative and matched/mismatched levels of stress on brain functioning during processing of emotional facial expressions.

The behavioral results did not show any relationship between early life stress, recent life stress, and their interaction in terms of accuracy and reaction time during performance of the face-matching task. This was not surprising, as the task was relatively easy and the presentation time of the stimuli was long enough to facilitate correct answers. Cumulative stress was a predictor of reaction time. Individuals with higher stress had shorter reaction times when processing negative emotional expression. This may be related to attentional bias to negative stimuli. Whole-brain fMRI analysis of the negative emotional expressions and neutral faces contrast for all participants revealed activations in the visual cortex, prefrontal cortex, and areas engaged in face perception (i.e., the FFA, OFA, and pSTS), which justifies the choice of these regions as seeds. Surprisingly, we did not observe activation of the amygdala. Nevertheless, these results are largely consistent with a model of brain activity relating face perception and expression processing (Duchaine & Yovel, 2015; Haxby et al., 2000).

We did not observe differences in whole-brain activation related to the match/mismatch model during the processing of negative emotional expressions, nor did we observe a relationship between the strength of activation and cumulative stress. This is surprising; however, there are some factors which could have led to such results, including the characteristics of both the sample and the stimuli. First, we used an overall index of negative experiences without distinguishing between types of negative life events – it has previously been shown that different stressors may have distinct impacts on functioning (Dragan, 2018; McLaughlin, & Sheridan, 2016). The intensity of emotional stimuli is also related to the brain response to these stimuli (Kensinger & Schacter, 2006; Silvers, Weber, Wager, & Ochsner, 2015). Given that emotional faces are less arousing than, for example, images depicting dramatic scenes or traumatic events (e.g., those that can be found in the International Affective Picture System; Alpers, Adolph, & Pauli, 2011; Britton, Taylor, Sudheimer, & Liberzon, 2006), it is possible that these stimuli did not pass the threshold of intensity for observation of a group difference. The choice of moderately arousing stimuli in our study could also be part of the reason that we did not observe whole-brain activation related to stress, and would similarly explain the lack of amygdala activation across all participants.

We found a main effect of early life stress on functional connectivity between the right amygdala and left central operculum as well as between the right FFA and bilateral supplementary motor area. Higher levels of stress were associated with weaker connectivity between these regions. This pattern of relations seems to be consistent with the results of previous studies showing deficits in facial expression processing in individuals with a history of early life stress (Curtis & Cicchetti, 2011; Lieslehto et al., 2017). Furthermore, higher levels of recent stress in adulthood were associated with stronger connectivity between the right pSTS and right insula. Different lines of evidence illustrate the impact of exposure to stress on insula reactivity and functional connections with this structure (Klumpp, Angstadt, & Phan, 2012; Li et al., 2014).

Our study found that both the interaction between early and recent life stress as well as cumulative stress levels were related to differences in functional connectivity in the brain during processing of negative emotional expressions. Seeds located in the FFA yielded some significant results for both models. In the match/mismatch model, the connections between the left FFA and right cerebellum were related to the interaction between early and recent life stress. In turn, the cumulative stress hypothesis explained differences in connectivity to regions associated with lower levels of visual processing, with higher levels of stress being associated with stronger anticorrelations between the right FFA and sLOC. Hermann, Bankó, Gál, and Vidnyánszky (2015) revealed that strength of resting-state functional connectivity between FFA and lateral occipital cortex (LOC) predicted the ability to discriminate the identity of noisy face images. Threat generalization is one of the consequences of experiencing extreme levels of stress (Lange et al., 2019; Morey et al., 2015) and altered activity of the LOC seems to be a part of the neural basis of this process (Lange et al., 2017). Negative association between cumulative stress index and connectivity between the FFA and sLOC probably reflect problems with emotional face discrimination. Differences in strengths of connections from the left and right OFAs also presented varying patterns depending on the model. Results were observed for the structure in the right hemisphere, which could be because this structure is characterized by functional asymmetry (Pitcher, Walsh, Yovel, & Duchaine, 2007).

The role of the OFA in face perception focuses on recognizing facial features (Pitcher, Walsh, & Duchaine, 2011), which is a step that precedes processing in the FFA (Duchaine & Yovel, 2015; Liu, Harris, & Kanwisher, 2010). The interaction between early and recent life stress was related to connectivity between the left OFA and regions of the right cerebellum as well as between the right OFA and left cerebellum, middle temporal gyrus, and supramarginal gyrus. The connectivity between the OFA and supramarginal gyrus may be related to paying attention to emotional expressions as the supramarginal gyrus has been found to be involved in the processing of emotionally salient stimuli (Santos, Mier, Kirsch & Meyer-Lindenberg, 2011; Vuilleumier et al., 2002).

It should be noted that our analysis revealed functional connections between both FFAs and the OFA and clusters in the parts of the cerebellum (right lobule VI, bilateral Crus I) that have previously been reported as being involved in emotional processing (Guell, Schmahmann, Gabrieli, & Ghosh, 2018; meta-analysis in Keren-Happuch, Chen, Ho, & Desmond, 2014). What does the functional coupling between structures involved in face processing and the cerebellum represent? How can we interpret the relationship between the interaction of early and recent life stress and the strength of this connectivity? It has been proposed (Doya, 1999) that one of the brain’s learning mechanisms (i.e., supervised learning) operates in the cerebellum. Supervised learning is when one neural network supplies instructive signals to another network. As a result, the instructed network learns to produce the desired output (Knudsen, 1994). Sokolov, Miall, and Ivry (2017) proposed that the cerebellum acts as a system that facilitates cortical processing. We could hypothesize that the detected functional connectivity reflects the prediction signal which the cerebellum sends to face-processing areas to promote their activity. The cerebellum possesses reciprocal connections with brain regions central to emotional processing (limbic system, prefrontal cortex; see review in Sokolov et al., 2017). It has been proposed that due to its physiological capacity, the cerebellum may integrate information from different brain areas, process it, and resend it to regions of interest (Snow, Stoesz, & Anderson, 2014). Thus, the functional coupling between face-processing areas and the cerebellum may represent the processes of integration of the emotional meaning of facial expressions. However, we must keep in mind that functional connectivity does not necessarily imply a causal relationship between two areas (Eickhoff & Müller, 2015). Therefore, there are other possible interpretations in which the cerebellum receives the input from FFAs and OFA or in which there exists exchange of information between these areas. In our study, participants who experienced low early life stress and high recent stress had weaker functional connectivity, while those who experienced high early life stress and high recent life stress had higher functional connectivity. Hence, keeping in mind the first interpretation of the functional connectivity, high levels of stress in both early and recent life may be associated with more “automated” processing of emotional facial expressions.

Cumulative stress explained more negative connectivity between the right OFA and the posterior MTG, which is involved in social cognition (interpreting others’ intentions; Mar, 2011). This is consistent with the result of the match/mismatch model in which participants who experienced low levels of early and recent life stress exhibited stronger connectivity between the right OFA and left middle temporal gyrus. Our findings are partially strengthened by the results of the study by Lei et al. (2018). They demonstrated reduced connectivity-amplitude coupling in the left MTG among patients with borderline personality disorder, characterized by high levels of early life stress. The significant cluster in the precentral/postcentral gyri is an interesting result. These regions are usually not mentioned in the context of emotional face processing, but some evidence suggests that somatosensory regions are also important for recognizing emotions from facial expressions through generating somatosensory representations of how the other individual feels (Adolphs, Damasio, Tranel, Cooper, & Damasio, 2000; Kragel & LaBar, 2016). It may be that the match/mismatch model and the cumulative model explain connections between the OFA and structures responsible for different aspects of emotional processing. Alternatively, it may be that the somatosensory regions are indeed not crucial for emotion processing and the increasingly negative correlations represent their higher disengagement in individuals who have experienced more stress and hence focus more attention on emotional processing.

The functional connectivity of pSTS also exhibited patterns only for the cumulative model. A negative correlation was found between the activity of the left pSTS and the activity of some of the areas in the default mode network, the activity of which is normally anticorrelated with goal-oriented actions (Uddin, Kelly, Biswal, Castellanos, & Milham, 2009). This may mean that people who have experienced higher cumulative stress show a greater attentional shift towards the performance of the facial expression processing task.

In summary, in the match/mismatch model, the interaction between early and recent stress was exhibited in functional connectivity of face-selective areas with the cerebellum, which is engaged in supervised learning processes. This may indicate that the interaction between early and recent life stress is characterized by more effective learning of the emotional value of encountered stimuli. Together, this may mean that emotional expression processing requires the stronger engagement of areas typically associated with emotion processing in those who experience only early or recent life stress, while it is more “automatic” in the presence of both early and recent stress. This interpretation would indicate that the matched stress has an advantage over mismatched early and recent life stress in emotional expression processing, which would be in line with the idea behind the match/mismatch hypothesis – that matched stress facilitates the action of more adaptive mechanisms which increase the fitness of an individual under adverse conditions (Nederhof & Schmidt, 2012). In turn, the negative correlations in the cumulative stress model may suggest that individuals who have experienced more overall stress devote more resources to emotional expression processing – their attention is more selectively focused on the task, which is reflected by suppressed activity in regions that are not crucial for these processes. This may influence the processing of external salient information, which takes the form of attentional bias towards threats (Pine et al., 2005), as supported by the shorter reaction time of individuals with higher cumulative stress levels. This altered processing of emotional stimuli may lead to the development of stress-related psychopathology (Jenness, Hankin, Young, & Smolen, 2016). Numerous studies have shown that stress-related disorders are associated with attentional bias towards emotional stimuli, which is evident especially in depression and anxiety disorders (Bar-Haim, Lamy, Pergamin, Bakermans-Kranenburg, & van IJzendoorn, 2007; Beck, 2008).

However, the proposed interpretations of the results should be considered with caution, as stress-related alterations in brain activity when performing tasks related to emotion processing can be explained in two ways: as a sign of a deficit or of compensation. Weaker activity or connections could indicate either ineffective involvement of the brain structures in question, or conversely, more efficient performance (Etkin, Prater, Hoeft, Menon, & Schatzberg, 2010; Phillips et al., 2008). On the other hand, higher activations or stronger connections could indicate more efficient processing, but could also mean that more cognitive and neural resources are needed to effectively perform the task (Etkin & Schatzberg, 2011; Reuter-Lorenz & Cappell, 2008).

To our knowledge, there has been no previous attempt to simultaneously explore models based on both the cumulative stress hypothesis and the match/mismatch hypothesis in the context of processing negative emotional expressions. Another strength is the use of neutral faces as the control condition, rather than shapes or objects. We were able to subtract neuronal activation related to processing emotional expressions, controlling the activation relating to face perception per se*.* However, the whole-brain analysis yielded activation in the fusiform gyrus, but not in the left and right amygdalae (which did not survive the FWE correction). Therefore, the use of neural faces as a control condition may not have fully isolated activation involved in the processing of emotional expressions as intended, but rather subtracted it out. One possible explanation is that neutral faces may still be more emotionally salient than geometric shapes, more often used in the face-matching paradigm.

We would also like to acknowledge some limitations of this study. First, resilience wasn’t measured in our study. Participants with high stress experience, but no psychopathology, may be a particularly resilient sample of adults. Future research should include resilience as it could potentially influence the results and elucidate the mechanisms behind match and mismatch effects and shed light on the levels of adaptation of different groups. Stress along with coping mechanisms determine one’s degree of resilience, defined as the ability to restore homeostasis and recover after impairment of one’s functioning (Franklin, Saab, & Mansuy, 2012; Karatsoreos & McEwen, 2011). Including resilience as a covariant would control for individual differences in resistance to adversities. The presence of psychiatric disorders as a part of exclusion criteria may be problematic given their association with early life stress (McLaughlin et al., 2010). We acknowledge that this is a potential limitation of our study; nevertheless, other studies indicated that differences in neurodevelopment may be confounded by psychiatric diagnoses (Hart & Rubia, 2012; Paquola, Bennett, & Lagopoulos, 2016). It is problematic to distinguish potential differences that are related to stress or to psychiatric diagnosis itself. Thus, we decided to follow the recommendations of Hart and Rubia (2012) and control for co-morbid psychopathology by including only individuals without psychiatric conditions. Our study also does not examine particular stressors separately; it is known that distinct adverse events (such as physical abuse, a death in the family, or being bullied) may have at least partially different consequences (Mclaughlin et al., 2016). These situations are characterized by differing quantities and qualities of deprivation and threat. Sheridan and McLaughlin (2014) suggest that these dimensions of adversities are characterized by different impact on neurodevelopment – deprivation leads to the development of a brain structure adapted to less complex environments, while threat affects neural networks involved in emotional processing. According to the match/mismatch model, early and recent life stress have similar significance (Nederhof & Schmidt, 2012). To the best of our knowledge, there are no studies that have directly compared the impact of particular stressors experienced at different periods of life on neurodevelopment. However, given the importance of timing of stressors in early life (Herzog & Schmahl, 2018), we cannot rule out the possibility that life stress experienced during childhood and adulthood can be quantitatively different, which may be a limitation of our study. It is also possible to distinguish between stressors related to personal experience and those related to other people (Dragan, 2018; Palgi, Shrira, Ben-Ezra, Shiovitz-Ezra, & Ayalon, 2012; Shmotkin & Litwin, 2009). Our study also did not differentiate between brief and chronic stress – both animal and human studies show that whether one develops psychopathology or resilience as a result of early stress may depend on the levels and duration of that stress (Franklin et al., 2012). Therefore, future studies on emotional processing should investigate how different adversities affect brain activations in the context of emotion processing. Lastly, previous studies focused on early adversities such as institutionalization or maltreatment. The most commonly reported stressors in our sample were events that are relatively low in severity, therefore direct comparison with previous results is difficult.

To conclude, we found that both the interaction between early life stress and recent life stress as well as cumulative stress levels were related to alterations in different neural circuits during emotion processing, observed as altered functional connectivity. Therefore, this research adds to the growing body of literature suggesting that models based on both the cumulative stress hypothesis and the match/mismatch hypothesis are useful for explaining the effects of stress on humans. Further studies are needed to explore the impacts of different kinds of stressors on functional connectivity during emotion processing and to determine whether the observed alterations in neural circuits are beneficial or maladaptive in the context of emotion processing.

## Electronic supplementary material

ESM 1 (PDF 533 kb)
