# Supplementary material for: The relationship between early and recent life stress and emotional expression processing: A functional connectivity study
Source: Cogn Affect Behav Neurosci. 2020 Apr 28;20(3):588–603. doi: 10.3758/s13415-020-00789-2 (PMC7266792; doi:10.3758/s13415-020-00789-2)
Supplement: Supplementary file 1 — (PDF 533 kb) [file 13415_2020_789_MOESM1_ESM.pdf]

Supplementary material for the article:

The Relationship Between Early and Recent Life Stress and Emotional Expression Processing—A Functional Connectivity Study

Andrzej Sokołowski[a], Monika Folkierska-Żukowska[a], Katarzyna Jednoróg[b], Craig A. Moodie[c], Wojciech Ł. Dragan[a]

[a]The Interdisciplinary Centre for Behavioural Genetics Research, Faculty of Psychology, University of Warsaw, Warsaw, Poland
[b]Laboratory of Language Neurobiology, Nencki Institute of Experimental Biology, Polish Academy of Sciences, Warsaw, Poland
[c]Department of Psychology, Stanford University, Stanford, USA

*Corresponding Author:
Wojciech Łukasz Dragan
Faculty of Psychology
University of Warsaw,
Warsaw, Poland
Postal address: Stawki 5/7, 00-183 Warszawa, Poland
Email: wdragan@psych.uw.edu.pl

Correspondence may also be addressed to Andrzej Sokołowski
Email: andrzej.sokolowski@ucsf.edu

Supplementary Material

ALE Meta-analysis.

Table S 1.
Studies concerning face perception and emotional expression processing included in the ALE meta-analysis.

	Coordinates (MNI)		
	x	y	z
Ambrus, Windel, Burton, & Kovács (2017)			
OFA R	42.64	-78.05	-8.47
OFA R	42.10	-77.60	-8.60
Axelrod & Yovel (2013)			
FFA L	-41	-59	-20
FFA R	40	-56	-20
pSTS L	-53	-57	8
pSTS R	52	-51	8
Axelrod & Yovel (2015)			
FFA L	-41	-53	-18
FFA R	39	-52	-16
pSTS L	-51	-54	11
pSTS R	52	-42	10
Beer et al. (2016)			
FFA R	37.69	-47.18	-21.85
Bilalić, Grottenthaler, Nägele, & Lindig (2016)			
FFA L	-45	-54	-21
FFA R	45	-54	-15
pSTS R	48	-36	0
Bona, Cattaneo, & Silvanto (2016)			
OFA R	39	-81	-10
Candidi, Stienen, Aglioti, & de Gelder (2015)			
pSTS R	56.37	-48.76	8.63
Carlin & Kriegeskorte (2017)			
FFA L	-39	-55	-20
FFA R	41.5	-50.5	-20.7
OFA L	-41.4	-82	-14.3
OFA R	43.5	-75.5	-12.6
Contreras, Banaji, & Mitchell (2013)			
FFA L	-37.1	-47.6	-17.3
FFA R	38.8	-44.3	-18.5
OFA L	-33.1	-77	-6.55
OFA R	33.3	-76.7	-8.9
pSTS L	-49.8	-52.8	21.3

pSTS R	49.8	-43.4	13.9
Davies-Thompson et al. (2018)			
FFA L	-42	-55	-26
FFA R	42	-58	-17
OFA L	-38	-78	-8
OFA R	39	-81	-11
pSTS L	-49	-55	15
pSTS R	51	-51	12
Engell, Kim, & McCarthy (2018)			
FFA R	44	-48	-22
OFA R	48	-76	-6
Goffaux, Schiltz, Mur, & Goebel (2013)			
FFA L	-40.08	-48.42	-19.28
FFA R	40.92	-45.04	-22.12
OFA L	-39.92	-75.65	-12.11
OFA R	44.36	-73.13	-12.67
Goffaux, Duecker, Hausfeld, Schiltz, & Goebel (2016)			
FFA L	-42.2	-51.41	-16.71
FFA R	41.02	-48.68	-15.04
OFA L	-39.74	-76.54	1.42
OFA R	40.16	-76.56	-3.3
Jiang, Badler, Righi, & Rossion (2015)			
FFA L	-41.12	-52.47	-16.62
FFA R	42.08	-54.3	-17.86
OFA L	-33.4	-85.2	-11.27
OFA R	34.72	-87.84	-8.81
pSTS R	54.24	-44.53	5.62
McGugin, Van Gulick, & Gauthier (2016)			
FFA L	-41.19	-63.93	-24.45
FFA L	-42.35	-43.73	-26.43
FFA R	44.18	-63.53	-24.83
FFA R	44.1	-41.09	-25.92
OFA L	-35.7	-86.25	-22.34
OFA R	32.39	-90.16	-21.99
Nguyen & Cunnington (2014)			
FFA L	-41	-51	-18
FFA R	41	-52	-16
OFA L	-40	-74	-13
OFA R	41	-73	-12
pSTS R	55	-55	11
Nichols, Betts, & Wilson (2016)			
FFA L	-37.89	-53.62	-17.68
FFA R	42.09	-53.13	-16.85

OFA L	-38.76	-78.32	-6.26
OFA R	45.5	-74.83	-8.04
Pajani, Kouider, Roux, & de Gardelle (2017)			
FFA L	-42	-51	-20
FFA R	43	-48	-20
OFA L	-42	-76	-11
OFA R	45	-73	-10
Pitcher (2014)			
OFA R	42	-79	-10
pSTS R	51	-70	2
Sauder, Hajcak, Angstadt, & Phan (2013)			
FFA L	-33	-60	-15
FFA L	-34	-62	-16
FFA R	33	-59	-15
FFA R	34	-60	-15
Slotnick & White (2013)			
FFA L	-38.98	-53.73	-18.78
FFA R	42.02	-47.06	-20.82
OFA L	-41.83	-77.09	7.12
OFA R	48.91	-73.58	5.22
pSTS L	-51.54	-57.35	12.05
pSTS R	52.17	-56.99	10.25
Sunday, McGugin, Tamber-Rosenau, & Gauthier (2018)			
FFA L	-41.62	-64.6	-19.2
FFA L	-42.22	-49.78	-22.3
FFA R	41.02	-64.8	-18.37
FFA R	40.72	-47.29	-23.89
OFA L	-39.41	-82.09	-15.87
OFA R	33.68	-82.85	-17.13
Bi, Chen, Zhou, He, & Fang (2014)			
FFA L	-43.26	-49.08	-14.68
FFA R	42.07	-48.88	-17.27
OFA L	-38.81	-75.44	-9.91
OFA R	40.06	-75.16	-11.28
pSTS L	-51.51	-55.98	15.28
pSTS R	52.17	-50.4	11.84
Weibert & Andrews (2015)			
FFA L	-42	-48	-24
FFA R	40	-52	-26
OFA L	-40	-82	-18
OFA R	48	-76	-8
pSTS L	-56	-60	6
pSTS R	46	-66	8

Yang et al. (2016)			
pSTS L	-57	-62	9
pSTS R	55	-59	7
Young et al. (2017)			
FFA L	-40	-51.6	-17.3
FFA R	40.9	-42.9	-18.4
Zachariou, Safiullah, & Ungerleider (2018)			
FFA L	-38.97	-48.21	-17.08
FFA R	44.21	-51.21	-19.32
OFA L	-44.09	-76.76	-0.72
OFA R	47.72	-76.54	-3.43
Zebrowitz, Ward, Boshyan, Gutchess, & Hadjikhani (2016)			
FFA R	42	-46	-26
Zhang et al. (2016)			
FFA R	44.14	-47.37	-24.18
OFA R	42.23	-78.35	-11
pSTS R	48.96	-45.84	14.8
Zhou et al. (2018)			
FFA L	-40	-53	-19
FFA R	42	-51	-18
OFA L	-40	-79	-11
OFA R	40	-77	-11

FFA–fusiform face area; L–left; OFA–occipital face area; pSTS–posterior superior temporal sulcus; R–right.

Whole-brain Analysis.

Table S 2.
Whole-brain analysis for the face-matching task across all participants (negative emotional expressions > neutral faces).

Brain region		x	y	z	T	k
**Emotional > Neutral**						
Fusiform gyrus	L	-40	-50	-16	12.17	6520
Inferior occipital gyrus	L	-30	-88	-6	11.37	lm
Inferior occipital gyrus	L	-40	-78	-8	11.22	lm
Inferior occipital gyrus	R	34	-86	-6	11.24	7345
Occipital fusiform gyrus	R	38	-64	-12	10.88	lm
Inferior occipital gyrus	R	48	-74	-4	10.33	lm
Inferior frontal gyrus	R	44	12	26	9.94	4604
Precentral gyrus	R	58	14	40	8.41	lm
Precentral gyrus	R	50	4	52	8.29	lm
Precentral/inferior frontal gyrus	L	-38	4	28	8.68	4174
Middle frontal gyrus	L	-48	14	32	8.13	lm
Middle frontal gyrus	L	-54	28	28	7.80	lm
Paracingulate gyrus/SMA	L	-6	12	50	6.89	594
**Neutral > Emotional**						
Posterior cingulate gyrus	R	2	-30	42	7.82	12076
Paracingulate gyrus	L	-2	54	2	7.34	lm
Paracingulate gyrus/ACC	R	-10	48	6	6.83	lm
Angular gyrus	R	60	-54	36	6.35	669
Angular gyrus	R	60	-52	44	6.01	lm
Middle occipital gyrus	R	48	-72	30	5.15	lm
sLOC	L	-42	-80	34	6.17	709
Angular gyrus	L	-52	-58	36	4.28	lm
Planum polare	R	58	-2	2	5.83	1210
Middle temporal gyrus	R	66	-16	-14	5.09	lm
Superior temporal gyrus	R	64	-14	4	4.76	lm
Planum temporale	L	-50	-34	12	4.59	805
Superior temporal gyrus	L	-62	-20	4	4.51	lm
Heschl's gyrus	L	-54	-8	0	4.17	lm

Coordinates in MNI space; L–left hemisphere; lm–local maximum; R–right hemisphere. ACC–anterior cingulate cortex; sLOC–superior lateral occipital cortex; SMA–supplementary motor area. $p < .001$ (unc.); FWE < .05.

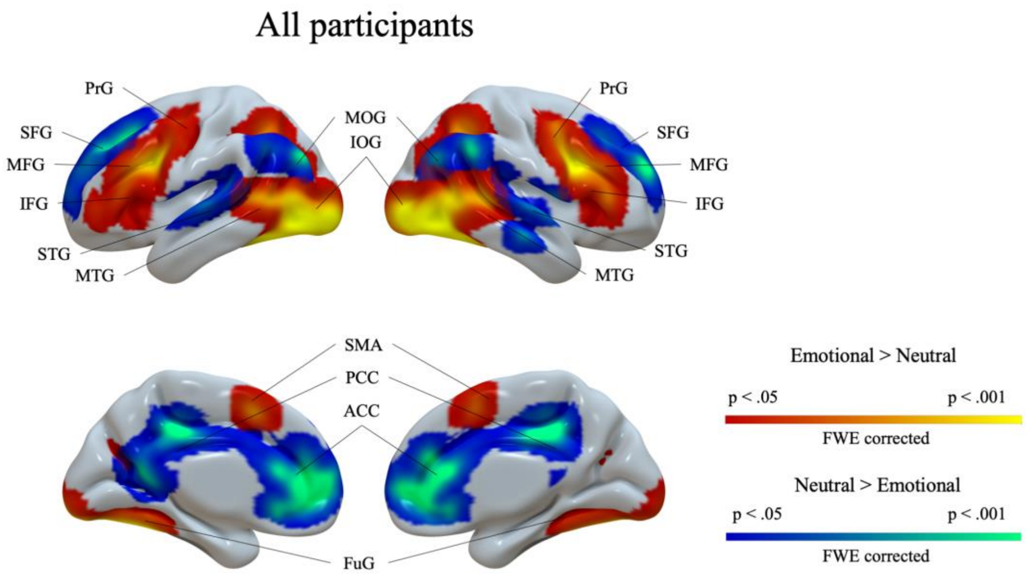

Figure S1. Whole-brain analysis across all participants (negative emotional expressions > neutral faces). Warm colors indicate stronger activation for emotional expressions; cool colors indicate deactivation. Abbreviations: ACC–anterior cingulate cortex; FuG–fusiform gyrus; IFG–inferior frontal gyrus; IOG–inferior occipital gyrus; MFG–middle frontal gyrus; MOG–middle occipital gyrus; MTG–middle temporal gyrus; PCC–posterior cingulate cortex; PrG–precentral gyrus; SFG–superior frontal gyrus; SMA–supplementary motor area; STG–superior temporal gyrus.

References

Ambrus, G. G., Windel, F., Burton, A. M., & Kovács, G. (2017). Causal evidence of the involvement of the right occipital face area in face-identity acquisition. *NeuroImage, 148,* 212–218. doi:10.1016/j.neuroimage.2017.01.043

Axelrod, V., & Yovel, G. (2013). The challenge of localizing the anterior temporal face area: A possible solution. *NeuroImage, 81,* 371–380. doi:10.1016/j. neuroimage.2013.05.015

Axelrod, V., & Yovel, G. (2015). Successful Decoding of Famous Faces in the Fusiform Face Area. *PLOS ONE*, *10*(2), e0117126. doi:10.1371/journal.pone.0117126

Beer, J. C., Smith, A. R., Jarcho, J. M., Chen, G., Reynolds, R. C., Pine, D. S., & Nelson, E. E. (2016). Anxiously elaborating the social percept: Anxiety and age differences in functional connectivity of the fusiform face area in a peer evaluation paradigm. *Australian Journal of Psychology*, *68*(3), 154–165. doi:10.1111/ajpy.12130

Bi, T., Chen, J., Zhou, T., He, Y., & Fang, F. (2014). Function and Structure of Human Left Fusiform Cortex Are Closely Associated with Perceptual Learning of Faces. *Current Biology*, *24*(2), 222–227. doi:10.1016/j.cub.2013.12.028

Bilalić, M., Grottenthaler, T., Nägele, T., & Lindig, T. (2016). The Faces in Radiological Images: Fusiform Face Area Supports Radiological Expertise. *Cerebral Cortex, 26*(3), 1004–1014. doi:10.1093/cercor/bhu272

Bona, S., Cattaneo, Z., & Silvanto, J. (2016). Investigating the Causal Role of rOFA in Holistic Detection of Mooney Faces and Objects: An fMRI-guided TMS Study. *Brain Stimulation*, *9*(4), 594–600. doi:10.1016/j.brs.2016.04.003

Candidi, M., Stienen, B. M. C., Aglioti, S. M., & de Gelder, B. (2015). Virtual lesion of right posterior superior temporal sulcus modulates conscious visual perception of fearful expressions in faces and bodies. *Cortex, 65*, 184–194. doi:10.1016/j.cortex.2015.01.012

Carlin, J. D., & Kriegeskorte, N. (2017). Adjudicating between face-coding models with individual-face fMRI responses. *PLOS Computational Biology*, *13*(7), e1005604. doi:10.1371/journal.pcbi.1005604

Contreras, J. M., Banaji, M. R., & Mitchell, J. P. (2013). Multivoxel Patterns in Fusiform Face Area Differentiate Faces by Sex and Race. *PLoS ONE, 8*(7), e69684. doi:10.1371/journal.pone.0069684

Davies-Thompson, J., Elli, G. V, Rezk, M., Benetti, S., van Ackeren, M., & Collignon, O. (2018). Hierarchical Brain Network for Face and Voice Integration of Emotion Expression. *Cerebral Cortex*, *bhy240*, 1–16. doi:10.1093/cercor/bhy240

Engell, A. D., Kim, N. Y., & McCarthy, G. (2018). Sensitivity to Faces with Typical and Atypical Part Configurations within Regions of the Face-processing Network: An fMRI Study. *Journal of Cognitive Neuroscience*, *30*(7), 963–972. doi:10.1162/jocn_a_01255

Goffaux, V., Duecker, F., Hausfeld, L., Schiltz, C., & Goebel, R. (2016). Horizontal tuning for faces originates in high-level Fusiform Face Area. *Neuropsychologia*, *81,* 1–11. doi:10.1016/j. neuropsychologia.2015.12.004

Goffaux, V., Schiltz, C., Mur, M., & Goebel, R. (2013). Local Discriminability Determines the Strength of Holistic Processing for Faces in the Fusiform Face Area.

Frontiers in Psychology, *3*(604), 1–14. doi:10.3389/fpsyg.2012.00604

Jiang, F., Badler, J. B., Righi, G., & Rossion, B. (2015). Category search speeds up face-selective fMRI responses in a non-hierarchical cortical face network. *Cortex*, *66*, 69–80. doi:10.1016/j.cortex.2015.01.025

McGugin, R. W., Van Gulick, A. E., & Gauthier, I. (2016). Cortical Thickness in Fusiform Face Area Predicts Face and Object Recognition Performance. *Journal of Cognitive Neuroscience*, *28*(2), 282–294. doi:10.1162/jocn_a_00891

Nguyen, V. T., & Cunnington, R. (2014). The superior temporal sulcus and the N170 during face processing: Single trial analysis of concurrent EEG–fMRI. *NeuroImage*, *86*, 492–502. doi:10.1016/j. neuroimage.2013.10.047

Nichols, D. F., Betts, L. R., & Wilson, H. R. (2016). Position selectivity in face-sensitive visual cortex to facial and nonfacial stimuli: an fMRI study. *Brain and Behavior*, *6*(11), e00542. doi:10.1002/brb3.542

Pajani, A., Kouider, S., Roux, P., & de Gardelle, V. (2017). Unsuppressible Repetition Suppression and exemplar-specific Expectation Suppression in the Fusiform Face Area. *Scientific Reports*, *7*, 160. doi:10.1038/s41598-017-00243-3

Pitcher, D. (2014). Facial Expression Recognition Takes Longer in the Posterior Superior Temporal Sulcus than in the Occipital Face Area. *Journal of Neuroscience*, *34*(27), 9173–9177. doi:10.1523/jneurosci.5038-13.2014

Sauder, C. L., Hajcak, G., Angstadt, M., & Phan, K. L. (2013). Test-retest reliability of amygdala response to emotional faces. *Psychophysiology*, *50*(11), 1147–1156. doi:10.1111/psyp.12129

Slotnick, S. D., & White, R. C. (2013). The fusiform face area responds equivalently to faces and abstract shapes in the left and central visual fields. *NeuroImage*, *83*, 408–417. doi:10.1016/j. neuroimage.2013.06.032

Sunday, M. A., McGugin, R. W., Tamber-Rosenau, B. J., & Gauthier, I. (2018). Visual imagery of faces and cars in face-selective visual areas. *PLOS ONE*, *13*(9), e0205041. doi:10.1371/journal.pone.0205041

Weibert, K., & Andrews, T. J. (2015). Activity in the right fusiform face area predicts the behavioural advantage for the perception of familiar faces. *Neuropsychologia*, *75*, 588–596. doi:10.1016/j. neuropsychologia.2015.07.015

Yang, Z., Zhen, Z., Huang, L., Kong, X., Wang, X., Song, Y., & Liu, J. (2016). Neural Univariate Activity and Multivariate Pattern in the Posterior Superior Temporal Sulcus Differentially Encode Facial Expression and Identity. *Scientific Reports*, *6*, 23427. doi:10.1038/srep23427

Young, L. R., Yu, W., Holloway, M., Rodgers, B. N., Chapman, S. B., & Krawczyk, D. C. (2017). Amygdala activation as a marker for selective attention toward neutral faces in a chronic traumatic brain injury population. *Neuropsychologia*, *104*, 214–222. doi:10.1016/j.neuropsychologia.2017.08.026

Zachariou, V., Safiullah, Z. N., & Ungerleider, L. G. (2018). The Fusiform and Occipital Face Areas Can Process a Nonface Category Equivalently to Faces. *Journal of Cognitive Neuroscience*, *30*(10), 1499–1516. doi:10.1162/jocn_a_01288

Zebrowitz, L., Ward, N., Boshyan, J., Gutchess, A., & Hadjikhani, N. (2016). Dedifferentiated face processing in older adults is linked to lower resting state metabolic

activity in fusiform face area. *Brain Research*, *1644*, 22–31. doi:10.1016/j.brainres.2016.05.007

Zhang, H., Japee, S., Nolan, R., Chu, C., Liu, N., & Ungerleider, L. G. (2016). Face-selective regions differ in their ability to classify facial expressions. *NeuroImage*, *130*, 77–90. doi:10.1016/j.neuroimage.2016.01.045

Zhou, G., Liu, J., Xiao, N. G., Wu, S. J., Li, H., & Lee, K. (2018). The Fusiform Face Area Plays a Greater Role in Holistic Processing for Own-Race Faces Than Other-Race Faces. *Frontiers in Human Neuroscience*, *12*, 220. doi:10.3389/fnhum.2018.00220
